# The Role of Helper T Cells in Psoriasis

**DOI:** 10.3389/fimmu.2021.788940

**Published:** 2021-12-15

**Authors:** Peng Hu, Mengyao Wang, Hu Gao, Ai Zheng, Jinhui Li, Dezhi Mu, Jiyu Tong

**Affiliations:** ^1^ West China Second University Hospital, Sichuan University, Chengdu, China; ^2^ Department of Pediatrics, West China Second University Hospital, Sichuan University, Chengdu, China; ^3^ Key Laboratory of Obstetrics & Gynecologic and Pediatric Diseases and Birth Defects of the Ministry of Education, Sichuan University, Chengdu, China; ^4^ Key Laboratory of Birth Defects and Related Diseases of Women and Children, Sichuan University, Ministry of Education, Chengdu, China; ^5^ Department of Emergency, West China Second University Hospital, Sichuan University, Chengdu, China; ^6^ Department of Immunology, West China School of Basic Medical Sciences and Forensic Medicine, Sichuan University, Chengdu, China

**Keywords:** Th17, Tregs, psoriasis, cytokines, biologics

## Abstract

Psoriasis is a complex, chronic relapsing and inflammatory skin disorder with a prevalence of approximately 2% in the general population worldwide. Psoriasis can be triggered by infections, physical injury and certain drugs. The most common type of psoriasis is psoriasis vulgaris, which primarily features dry, well-demarcated, raised red lesions with adherent silvery scales on the skin and joints. Over the past few decades, scientific research has helped us reveal that innate and adaptive immune cells contribute to the chronic inflammatory pathological process of psoriasis. In particular, dysfunctional helper T cells (Th1, Th17, Th22, and Treg cells) are indispensable factors in psoriasis development. When stimulated by certain triggers, antigen-presenting cells (APCs) can release pro-inflammatory factors (IL-23, IFN-α and IL-12), which further activate naive T cells and polarize them into distinct helper T cell subsets that produce numerous cytokines, such as TNF, IFN-γ, IL-17 and IL-22, which act on keratinocytes to amplify psoriatic inflammation. In this review, we describe the function of helper T cells in psoriasis and summarize currently targeted anti-psoriatic therapies.

## Introduction

Psoriasis is a complex, chronic relapsing and inflammatory skin disorder with an overall prevalence of 2% in the general population worldwide (1). The most common type of psoriasis is psoriasis vulgaris, which primarily manifests as dry, well-demarcated, raised red lesions with adherent silvery scales on the skin and joints and accounts for nearly 90% of all psoriasis cases. Psoriasis is also associated with multiple comorbidities, such as arthritis, obesity, diabetes mellitus, depression, hypertension, cardiovascular disease, and reduced quality of life (2).

Although the exact mechanism that triggers psoriasis remains unclear, it is currently accepted that psoriasis is induced or exacerbated by either nonspecific triggers, such as infections [such as Streptococcus (3)], physical injury [such as scratching and tattoos (4)], drugs [such as β blockers, lithium and antimalarials (5, 6)] or some specific autoantigens [such as cathelicidin LL-37, melanocytic ADAMTSL5, lipid antigen PLA2G4D and keratin 17 (7)]. Pathologically, psoriasis is characterized by epidermal acanthosis (thickening of the viable layers), hyperkeratosis (thickened cornified layer), and parakeratosis (cell nuclei present in the cornified layer) (8). Over the past 50 years, researchers have performed substantial work to explore the underlying mechanism of the link between skin injury and keratinocyte dysfunction, which drives the development and progression of psoriasis. A series of basic and clinical studies has shown that psoriasis is mediated by components of both the innate and adaptive immune systems. It was reported that innate immune cells such as natural killer (NK) cells, NKT cells, neutrophils, mast cells, γδ T cells, and dendritic cells (DCs) were significantly increased in psoriatic lesions and could frequently release pathogenic mediators such as TNF-α and interleukin 23 (IL-23) (9–13). As another important source of cytokines, adaptive immune cells have been the subject of academic interest since 1979. A variety of studies showed that several related cytokines, such as tumor necrosis factor-α (TNF-α), interferon γ (IFN-γ), interleukin 23 (IL-23), interleukin 17 (IL-17), and interleukin 22 (IL-22), were highly correlated with psoriasis. Recently, autoreactive T cells against specific autoantigens were also found to produce related pathogenic cytokines, especially IFN-γ and IL-17. Lande et al. demonstrated specific CD4 and CD8 T-cell responses and increased IFN-γ and IL-17 production to LL37 in psoriatic patients (14), while Arakwa et al. identified ADAMTSL5 as an autoantigen recognized by specific CD8 T-cells (15). Specially, for CD8 T cells, both autoantigens were showed to be presented in the peptide-binding groove of the human leukocyte antigen (HLA)-class I molecule encoded by the major psoriasis risk gene, HLA-Cw*06:02 (14, 15). Consistently, keratin peptides that share sequences with Streptococcal M-protein can be recognized by T cells from psoriatic patients (16–18). Subsequent evidences showed that full-length keratin 17 and its peptide fragments induce T cell proliferation and IFN-γ production, particularly in patients with the HLA-Cw*06:02 allele (19, 20). Furthermore, clinic studies showed that matched biological agents against these cytokines could also induce effective therapeutic results. Moreover, among them, IL-17, which is mainly produced by γδ T cells, CD4+ helper T cells (Th17 cells), and CD8+ cytotoxic T cells (Tc17 cells), seems to be most strongly implicated in psoriasis; thus, T cells, especially helper T (Th) cells, have become a hot topic in psoriasis pathogenesis. Here, we reviewed the biogenesis and function of helper T cells in psoriasis and briefly summarized currently targeted therapies.

## Role of Helper T Cells in Psoriasis

**Figure 1:**
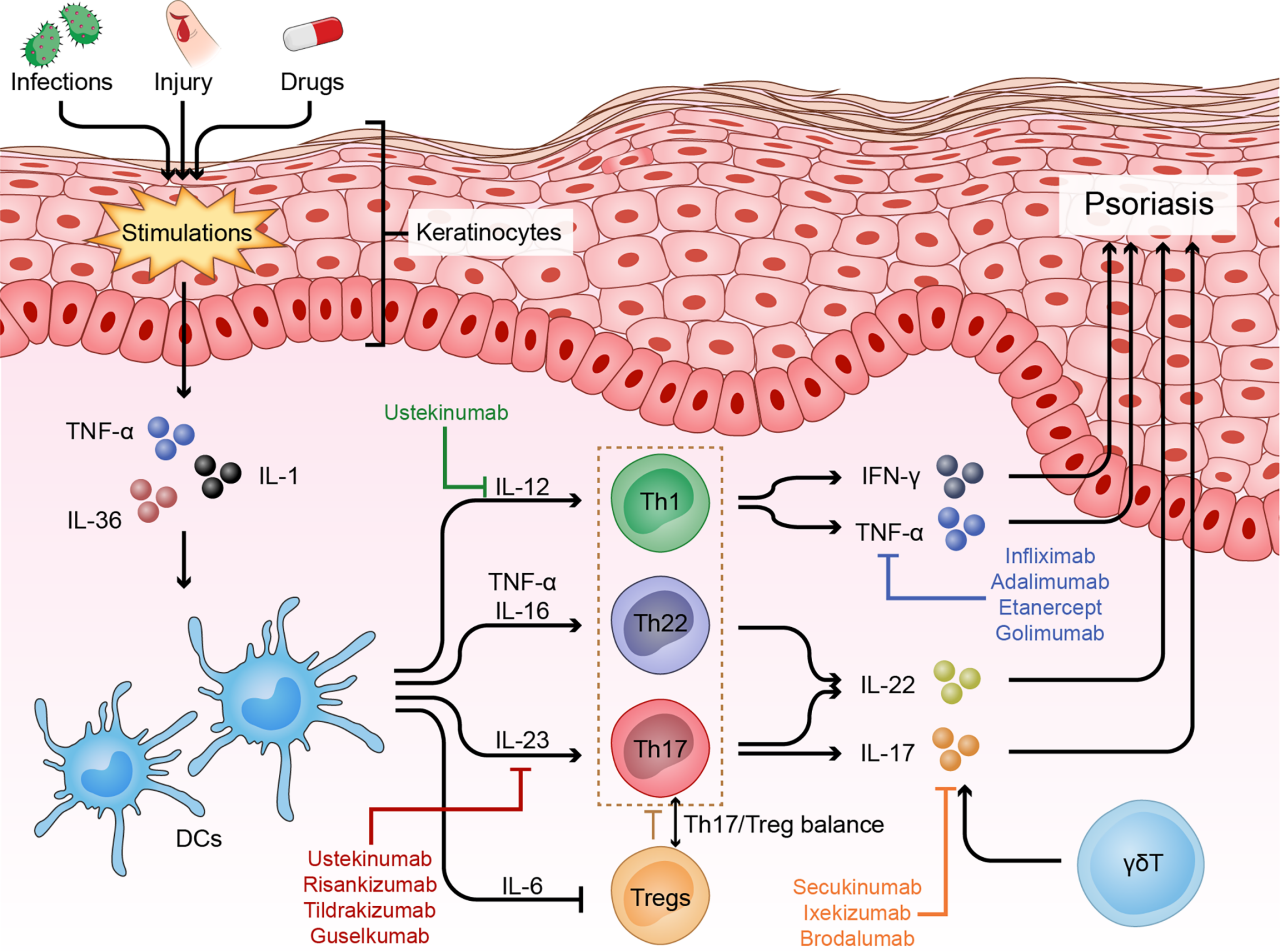
Immune dysfunction of psoriasis. Psoriasis is driven by many nonspecific triggers. Triggers such as infections and physical injury stimulate DCs to release pro-inflammatory factors (IL-23, TNF-α and IL-12). These cytokines in turn activate the IL-23 and/or IL-22 pathway to induce Th17 and/or Th22 cell differentiation, resulting in the production of numerous psoriatic cytokines, such as TNF-α, IFN-γ, IL-17 and IL-22, which act on keratinocytes to amplify psoriatic inflammation. In addition, skin infiltrating cells, such as γδT cells, contribute to the disease development *via* producing IL-17, and Treg cells and the Th17/Treg balance also play important roles in the pathogenesis of psoriasis.

When Mueller et al. used cyclosporine A, an immunosuppressive agent that inhibits T cell proliferation and cytokine production, to treat psoriasis and then observed surprising therapeutic efficacy, researchers realized the potential role of T cells in psoriasis pathogenesis (21). Later, Prinz et al. isolated 10 T cell lines and 105 T cell clones from the dermis and epidermis of psoriatic skin specimens and subsequently found that T cells and their secreted products, such as IFN-γ, could contribute to keratinocyte proliferation (22). Then, Baker et al. showed that the initial phase of psoriasis was dominated by epidermal infiltration of activated CD4+ T cells, indicating a primary immune trigger for the inflammatory and hyper-proliferative processes (23). In the current model, the crosstalk between keratinocytes and various immune cells, especially helper T cells, plays a central role in the progression of psoriasis (**Figure 1**).

### Th1/Th17/Th22 Cells in Psoriasis

A study from the University of Mainz investigated cytokines secreted by T cells obtained from epidermal specimens of psoriatic patients and showed that nearly all T cells tested produced Th1-related cytokines (IFN-γ, TNF-α, and IL-2), whereas only a minority of cells secreted Th2-related cytokines (IL-4 and IL-10) (24). In the same year, another study suggested that IFN-γ, which is produced mainly by Th1 cells, is capable of enhancing keratinocyte proliferation *in vitro* (22). These results defined psoriasis as a Th1 cell-mediated disease. However, the administration of humanized monoclonal antibodies against IFN-γ and TNF-α does not significantly improve psoriasis, suggesting that Th1 cells or their related cytokines may not be critical in the pathogenesis of psoriasis (25).

While assessing the involvement of IL-23 in the induction and maintenance of chronic inflammatory diseases, Harrington et al. first recognized the distinct CD4+ T cells - known as Th17 cells (26, 27). IL-23, a heterodimeric cytokine composed of a unique p19 chain and a p40 chain that is shared with IL-12, is essential for the survival and development of Th17 cells (28). There is growing evidence to suggest that the IL-23/Th17 axis and the related cytokines have critical roles in psoriasis. Enhanced expression of IL-23 at the mRNA and protein levels could be detected in psoriatic skin compared with healthy skin. Moreover, intradermal injection of IL-23 in murine models can stimulate keratinocyte proliferation and cause epidermal hyperplasia, and anti-IL-12/IL-23 and anti-IL-23 agents have shown highly effective therapeutic effects in clinical trials (29–32). In 2007, Wilson et al. found that Th17 cells may participate in the pathological processes of psoriasis through the coordinated expression of IL-17A, IL-17F, IL-22, IL-21 and IL-26 (33). Both IL-17A and IL-17F are subtypes of IL-17, and they have been shown to be elevated in psoriatic lesions and the peripheral blood of psoriatic patients (34). Clinical randomized trials have shown beneficial effects of IL-17A and IL-17F antibodies, validating IL-17A and IL-17F as potential therapeutic targets (35–38). Further studies demonstrated that IL-22 also mediates IL-23/Th17 axis-induced psoriasis-like skin inflammation (39). Zheng et al. suggested that IL-22, which acts in synergy with IL-17, might play an essential role in the pathogenesis of autoimmune diseases such as psoriasis (39). Subsequently, scientific research highlighted that IL-17 and IL-22 may mediate distinct downstream pathways that contribute to the psoriatic phenotype: IL-17 is more pro-inflammatory than IL-22, while IL-22 impairs keratinocyte differentiation (40). In addition, Van Belle et al. showed that IL-22 played major roles not only in the development of pustules and acanthosis but also in neutrophil infiltration in a mouse model triggered by the Toll-like receptor (TLR) 7/8 agonist imiquimod (41). The IL-23/Th17 axis and the related cytokines could further amplify keratinocyte proliferation and cause epidermal hyperplasia (42).

Later, Nograles et al. discovered a new subtype of T cells by analyzing the T cell subsets in skin biopsies and peripheral blood collected from psoriatic patients by intracellular cytokine staining and flow cytometry, and these cells were called Th22 cells (43). Moreover, research from Trifari et al. solidified this view. The researchers identified another population of human helper T cells that produced abundant IL-22 and IL-13 but no IFN-γ, IL-4 or IL-17. IL-22, as we described above, is required for the pathogenesis and development of many autoimmune diseases (44). Kagami et al. analyzed T cell numbers in the circulation of psoriatic patients and showed that in addition to Th1 and Th17 cells, Th22 cells were also increased in psoriatic patients (45). Notably, IL-22 deficiency caused a significant decrease in epidermal acanthosis and dermal inflammation induced by IL-23 (39). These studies established the crucial role of the IL-23/Th17 axis and the IL-22/Th22 pathway in the pathogenesis and development of psoriasis. However, it has been reported that Th1 and Th17 cells may contribute to the pathogenesis and development of psoriasis. Researchers argued that IFN-γ secreted mainly by Th1 cells could induce Th17 cells *via* IL-1 and IL-23. Furthermore, IFN-γ can also stimulate antigen-presenting cells (APCs) to produce CCL20, which is responsible for the migration of IL-17+ T cells (46). Considering the role of IL-22, it can no longer be denied that psoriasis is a Th1/Th17/Th22-mediated autoimmune disease.

### Treg Cells in Psoriasis

Treg cells are a special subset of helper T cells that are characterized by high expression of the CD25, alpha-chain of IL-2 receptor. Then Foxp3 (Forkhead Box P3), a transcription factor of the fork head/winged-helix family, was found as the most important transcription factor for controlling the development and function of Treg cells. Treg cells can suppress the activities of other effector immune cells mainly by direct contact and/or the secretion of suppressive cytokines, such as IL-10 and transforming growth factor (TGF)-β (47). Hence, Treg cells are prominently associated with peripheral tolerance, autoimmune diseases and chronic inflammatory diseases, including psoriasis (48).

Studies investigating the percentage of Treg cells in lesional skin or peripheral blood have contradictory evidence, which varied in different psoriasis subtypes, disease states, Treg definition, and types of samples (49–55). Interestingly, the function of Treg cells was consistently demonstrated to be impaired (56). Both Sugiyama et al. and Li B et al. observed that circulating CD4+CD25+ Treg cells in psoriatic patients failed to suppress the proliferation of normal CD4+CD25- responder T cells during co-culture (56, 57). Similar results were reported in pediatric patients (58).

The unstable expression (downregulation) of Foxp3 reflects the dysfunction of Treg cells in psoriasis, and numerous upstream regulators have been reported. One of these regulators is the pro-inflammatory cytokine milieu, especially high levels of IL-6. Indeed, IL-6 was highly expressed in the plasma and lesional skin of psoriatic patients (59). In an *in vitro* model, Goodman et al. reported that human Treg cell-mediated suppression of responder T cell proliferation could be reversed by culture with rhIL6 (recombinant human IL-6) or activated DCs, which highly express IL-6 (59). Furthermore, under IL-6 stimulation, elevated phosphorylation of the transcription factor Stat3 was noted in dysfunctional Treg cells (60). Stat3 acts as a downstream molecule of IL-6R and can bind to a silencing element within the Foxp3 locus, leading to a reduction in the expression of Foxp3. Furthermore, Zhao et al. reported that increased expression of microRNA-210 in CD4+ T cells from patients with psoriasis vulgaris repressed Foxp3 expression and subsequently inhibited the production of IL-10 and TGF-β (61). In addition, in a CD18-knockout mouse model, a spontaneous psoriasiform phenotype was observed, accompanied by Treg cell dysfunction. Adoptive transfer of wild-type Treg cells into CD18-knockout mice markedly reduced the psoriasis area and severity index (PASI) scores. Wang et al. revealed that reduced CD18 expression on CD4+CD25+CD127– Tregs resulted in deceased expression of TGF-β1 by disrupting their cell-cell contact with DCs (62).

Because the differentiation of Th17 and Treg cells is reciprocally regulated by shared and different cytokines, it is not surprising that the cytokine milieu of the psoriatic skin microenvironment may cause an imbalance. Zhang et al. and Wang et al. independently reported a positive association between the ratio of Th17 cells to Treg cells in peripheral blood and PASI scores (51, 61). As mentioned previously, IL-6, which is a critical cytokine for Th17 differentiation, inhibits Treg cells by inhibiting the expression of Foxp3 in psoriasis pathogenesis (63). Furthermore, a group of IL-17A+/Foxp3+/CD4+ triple-positive cells were identified in the lesional skin of psoriasis patients (64). These cells maintain a high RORγt/Foxp3 ratio to promote the production of IL-17A. This evidence suggests important plasticity between Th17 and Treg cells in psoriasis (64). Specifically, Singh et al. reported a mechanism by which reduced CD18 levels could promote the conversion of Treg cells to Th17 cells in a CD18-knockout mouse model of psoriasis (65). In addition, Ma et al. showed that the Th17/Treg imbalance was also regulated by the Notch1 signaling pathway, which is known to be a conserved signaling pathway involved in cell development and differentiation of multiple organisms and tissues (66). In their experiments, the expression of Notch1 and its target gene Hes-1 were positively correlated with PASI scores and the ratios of Th17/Treg cells. Correspondingly, Notch receptor inhibitors reduced the percentage of Th17 cells and the Th17/Treg ratio (66).

Currently, it is relatively clear that Treg cells and the Th17/Treg balance play important roles in the pathogenesis of psoriasis; however, the underlying mechanisms that drive Treg cell dysfunction and the imbalance in Th17/Treg cells still need to be further investigated.

### T Follicular Helper Cells

T follicular helper (Tfh) cells, characterized by the high expression of chemokine CXC receptor 5 (CXCR5), are another specialized subset of CD4+ T cells. With expression of IL-21, CXCL13 and PD-1, they exert B helper activities in a manner primarily dependent on IL-21 (67). Tfh cells can be further classified as three subpopulations, including Type 17 (CXCR3-CCR6+), Type 1 (CXCR3 + CCR6-), and Type 2 (CXCR3-CCR6-) cells. Recently, Tfh cells have been shown to be involved in the pathogenesis of psoriasis. Niu et al. and Wang et al. demonstrated that the frequency of circulating Tfh were elevated in psoriasis and positively correlated with serum IL-21 levels and PASI scores (68, 69), suggesting Tfh cells as potential contributors to psoriasis pathogenesis. Moreover, Caruso et al. reported that increased IL-21 was produced primarily by CD4+ T cells in psoriatic lesions (70). Although other markers, like CXCR5, were not analyzed to define CD4+ T cell subsets, a part of the IL-21-producing CD4 T cells were co-producing IFN-γ or IL-17. Consistently, Wang et al. observed that CXCR3−CCR6+ Tfh type 17 subset, which secrete the Th17 cytokines IL-17A and IL-22, increased and correlated with PASI score in psoriasis (71).

## Targeted Psoriasis Therapies

Recognition of the IL-23/Th17 axis and IL-22/Th22 pathway vigorously promoted the development of targeted therapies. To date, targeted therapies that have been approved by the Food and Drug Administration (FDA) for psoriasis treatment have shown promising effects (72, 73). There are also various new biologic agents that exhibit promising therapeutic efficacy in clinical trials (72, 73). **Table 1** shows the targeted agents for psoriasis treatment, and we briefly discuss the agents targeting the IL-23/Th17 axis and IL-22/Th22 pathway in the treatment of psoriasis.

**Table 1:** US food and drug administration–approved biologic treatments for psoriasis.

Biologics	Drug	Main Trials (Reference)	N	Control Intervention	Efficacy (VS Control Intervention)	
					PASI 75	PASI 90
Anti-TNF	Etanercept	Papp et al. (74)	583	PBO	49% at week 12 (VS 3%)	21% at week 12 (VS 1%)
	Infliximab	Reich et al. (75)	378	PBO	80% at week 10 (VS 3%) 82% at week 24 (VS 4%) 61% at week 50 (VS N/A)	57% at week 10 (VS 1%) 58% at week 24 (VS 1%) 45% at week 50 (VS N/A)
		Barker et al. (76)	868	MTX	78% at week 16 (VS 42%) 76.9% at week 26 (VS 30.7%)	54.5% at week 16 (VS 19.1%) 51.0% at week 26 (VS 14.9%)
	Adalimumab	Menter et al. (77)	1212	PBO	71% at week 16 (VS 7%)	N/A
	Certolizumab pegol	Gottlieb et al. (78)	461	PBO	82% at week 16 (VS 9.9%) 83.6% at week 48 (VS N/A)	52.2% at week 16 (VS 2.5%)
		Lebwohl et al. (79)	224	Etanercept	66.7% at week 12 (VS 53.3%)	N/A
Anti-IL-23	Ustekinumab	Leonardi et al. (32)	511	PBO	66.4% at week 12 (VS 3.1%) 78.6% at week 28 (VS N/A)	36.7% at week 12 (VS 2.0%) 55.6% at week 28 (VS N/A)
		Papp et al. (80)	821	PBO	75.7% at week 12 (VS 3.7%) 78.5% at week 28 (VS N/A)	50.9% at week 12 (VS 0.7%) 54.3% at week 28 (VS N/A)
	Briakinumab	Gottlieb et al. (81)	347	Etanercept/PBO	81.9% at week 12 (VS 56.0%/7.4%)	N/A
		Strober et al. (29)	350	Etanercept/PBO	80.6% at week 12 (VS 39.6%/6.9%)	55.4% at week 12 (VS 13.7%/4.2%)
	Tildrakizumab	Reich et al. (31)	463	PBO	62% at week 12 (VS 6%)	35% at week 12 (VS 3%)
			783	Etanercept/PBO	66% at week 12 (VS 48%/6%)	37% at week 12 (VS 21%/1%)
	Guselkumab	Blauvelt et al. (82)	663	Adalimumab	91.2% at week 16 (VS 73.1%) 91.2% at week 24 (VS 72.2%) 87.8% at week 48 (VS 62.6%)	73.3% at week 16 (VS 49.7%) 80.2% at week 24 (VS 53.0%) 76.3% at week 48 (VS 47.9%)
		Reich et al. (83)	1048	Secukinumab	85% at week 48 (VS 80%)	84% at week 48 (VS 70%)
		Thaçi et al. (84)	119	FAE	90.0% at week 24 (VS 27.1%)	81.7% at week 24 (VS 13.6%)
	Risankizumab	Gordon et al. (30)	506	Ustekinumab/PBO	N/A	75.3% at week 16 (VS 42%/4.9%)
			491	Ustekinumab/PBO	N/A	74.8% at week 16 (VS 47.5%/2.0%)
		Reich et al. (85)	605	Adalimumab	91% at week 16 (VS 72%)	72% at week 16 (VS 47%)
		Warren et al. (86)	327	Secukinumab	92% at week 16 (VS 80%) 90% at week 52 (VS 70%)	74% at week 16 (VS 66%) 87% at week 52 (VS 57%)
Anti-IL-17	Secukinumab	Langley et al. (38)	493	PBO	81.6% at week 12 (VS 4.5%)	59.2% at week 12 (VS 1.2%)
			979	Etanercept/PBO	77.1% at week 12 (VS 44%/4.9%)	54.2% at week 12 (VS 20.7%/1.5%)
		Thaçi et al. (87)	676	Ustekinumab	93.1% at week 16 (VS 82.7%)	79.0% at week 16 (VS 57.6%)
		Blauvelt 2017 (88)	676	Ustekinumab	92.5% at week 24 (VS 83.6%) 91.6% at week 52 (VS 78.2%)	80.8% at week 24 (VS 66.3%) 74.9% at week 52 (VS 60.6%)
		Bagel et al. (89)	1102	Ustekinumab	89.0% at week 52 (VS 82.1%)	73.2% at week 52 (VS 59.8%)
	Ixekizumab	Griffiths et al. (90)	877	Etanercept/PBO	89.7% at week 12 (VS 41.6%/2.4%)	70.7% at week 12 (VS 18.7%/0.6%)
			960	Etanercept/PBO	87.3% at week 12 (VS 53.4%/7.3%)	68.1% at week 12 (VS 25.7%/3.1%)
		Gordon et al. (91)	864	PBO	89.1% at week 12 (VS 3.9%)	70.9% at week 12 (VS 0.5%)
			385	N/A	83% at week 60	73% at week 60
		Blauvelt et al. (92)	385	N/A	83.6% at week 108	70.3% at week 108
		Lebwohl et al. (93)	385	N/A	82.8% at week 204	48.3% at week 204
		Reich et al. (94)	162	MTX/FAE	91% at week 24 (VS 70%/22%)	80% at week 24 (VS 39%/9%)
		Blauvelt et al. (95)	1027	Guselkumab	23% at week 2 (VS 5%) No statistically difference at week 24	58% at week 8 (VS 36%) No statistically difference at week 24
	Brodalumab	Lebwohl et al. (96)	1221	Ustekinumab/PBO	86% at week 12 (VS 70%/8%)	N/A
		Papp et al. (36)	1252	Ustekinumab/PBO	85% at week 12 (VS 69%/6%)	N/A
			441	PBO	83.3% at week 12 (VS 2.7%)	70.3% at week 12 (VS 0.9%)
		Pinter et al. (97)	149	FAE	81.0% at week 24 (VS 38.1%)	65.7% at week 12 (VS 21.9%)

TNF, tumor necrosis factor; N, Number of participants; PASI 75/90, a 75%/90% reduction in Psoriasis Area Severity Index (PASI) score compared with baseline; N/A, Not Applicable; MTX, Methotrexate; FAE, fumaric acid esters; PBO, Placebo.

### Targeting IL-23

IL-23 is a heterodimeric cytokine composed of a unique p19 chain and a p40 chain shared with IL-12. Targeted drugs include antibodies that inhibit the p19 subunit of IL-23 and the p40 subunit of both IL-12 and IL-23. Ustekinumab and Briakinumab, which are human interleukin-12/23 monoclonal antibodies, have been approved by the FDA and have shown significant efficacy in patients with moderate to severe plaque psoriasis (29, 32). Risankizumab and Tildrakizumab are agents that target the unique IL-23 subunit p19. The results of randomized controlled, phase 3 trials showed that these agents have superior efficacy to placebo or Ustekinumab in the treatment of moderate-to-severe plaque psoriasis (30, 31). Data from clinical trials have shown that IL-23 inhibitors have convincing safety profiles; however, long-term observations indicate that adverse events still occur during the treatment. A multicentre, randomized, double-blind, placebo-controlled clinical trial of Ustekinumab showed that adverse events were observed in 217 (53·1%) patients in the 45 mg group, 197 (47·9%) in the 90 mg group and 204 (49·8%) in the placebo group (80). The most common adverse effects included infections, headache and injection-site reactions (98).

### Targeting IL-17

Additional categories of biologic agents include fully human interleukin-17A/F monoclonal antibodies. Secukinumab and Ixekizumab can directly neutralize IL-17A and have excellent and sustained efficacy for psoriatic patients with or without PsA and nail psoriasis (37, 38, 90, 99–101). Bimekizumab, a human anti-IL-17 drug that inhibits both IL-17A and IL-17F, induced convincing clinical improvements in psoriatic patients with or without psoriatic arthritis (35, 102). Brodalumab is a human anti-IL-17 receptor antagonist (IL-17RA) (36). The feature that broadly blocks IL-17A, IL-17F, IL-17C and IL-17E enables IL-17RA to rapidly improve the clinical and histological features of psoriasis (103). It was shown that Brodalumab may even be effective in those who failed to respond to Secukinumab and Ixekizumab (103, 104). Although all these antagonists showed robust efficacy, unexpected side effects have also been reported. The most common adverse effects are infections, such as bacterial infection and tuberculosis; others, such as headache and diarrhea, are less common (73).

### Targeting IL-22

IL-22 is an essential cytokine for psoriasis development. ILV-095, an IL-22 receptor antagonist that was developed to treat psoriasis, failed in a phase I clinical trial because the expected endpoints could not be met (105). Another humanized monoclonal antibody against the IL-22 receptor, IL-094, was suspended for similar reasons (105). Thus far, biological therapies against the IL-22 receptor need to be further investigated in the future.

### Targeting JAK/STAT Pathway

The Janus kinase (JAK) signal transducer and activator of transcription (STAT) signaling pathway (JAK/STAT pathway) transduce signals from multitudes of cytokines and growth factors and plays a major role in the pathogenesis of many inflammatory and autoimmune diseases. Due to the essential role in forwarding the IL-23/Th17 axis and IL-22/Th22 pathway signals into cell, JAK/STAT pathway has received increasing attention recently in psoriasis (106). Furthermore, it is an attractive idea that to blockade multiple psoriasis related cytokines rather than specific to one signaling pathway by inhibiting of JAKs. Indeed, there are already several biologic agents that target JAK/STAT pathway in clinical trials: Tofacitinib (NCT01241591) for JAK1 and JAK3 (107); Baricitinib (NCT01490632) for JAK1 and JAK2 (108); Itacitinib (NCT01634087) and Solcitinib (NCT01782664) for JAK1 (109, 110); PF-06700841 (NCT02969018) for JAK1 and TYK2 (111); and PF-06826647 (NCT03895372) and Deucravacitinib (NCT03924427) for TYK2 (112, 113). Many of them have shown promising efficacy, especially selective TYK2 inhibitors, whereas the adverse effect and long-term safety still need to be emphasized.

## Conclusions

Over the past 50 years, substantial scientific research has suggested that T cells are closely associated with the pathogenesis of psoriasis. These cells serve as a link connecting nonspecific triggers and keratinocyte dysfunction. Triggers such as infections and physical injury stimulate antigen-presenting cells (APCs) to release pro-inflammatory factors (IL-23, IFN-α and IL-12). These cytokines in turn activate the IL-23 and/or IL-22 pathway to induce Th17 and/or Th22 cell differentiation, resulting in the production of numerous psoriatic cytokines, such as TNF, IFN-γ, IL-17 and IL-22, which act on keratinocytes to amplify psoriatic inflammation. Nowadays, on the one hand, the observation that Treg and/or Th17/Treg balance is frequently dysregulated in psoriatic patients hints at the critical role of Treg cells in controlling psoriatic inflammation. One possible mechanism by which the Th17/Treg balance is disturbed in psoriatic lesions attributes to the high levels of IL-6. And Tfh cells, which has been proved to be one of the major sources of IL-21, are also involve in the pathogenesis of psoriasis. Additionally, it has been reported that γδT cells, which are the major IL-17-producing cells in the skin, also play critical roles in psoriasis pathogenesis. On the other hand, biological agents targeting TNF, IL-23 and IL-17 have shown promising efficacy during the clinical treatment of psoriasis, while IL-6 inhibitors, IL-21 inhibitors and recombinant human IL-10 treatment didn’t attain the results as expected. Moreover, many clinical trials are ongoing to uncover new targets in psoriasis. For example, JAK inhibitors paved the way of inhibiting multiple pro-inflammatory cytokines together and have shown clinical efficacy in both phase II and III trials. All these advancements are ascribed to the continuous exploration of the pathogenesis of psoriasis. However, there are still some issues that need to be addressed. First, adverse events cannot be ignored during treatment, which may be feasibly due to the broad biological effects of these cytokines. In addition, several limitations, such as the difficulty of effecting a cure, the need for long-term medication and ease of recurrence after drug withdrawal, still exist. All this evidence highlights that our understanding of psoriasis is insufficient, especially the crosstalk of multiple immune cells and cytokines in psoriasis, which still needs to be studied in depth. Further studies should focus on identifying current therapeutic approaches and novel, efficient targets to help overcome the adverse effects and limitations.

## Author Contributions

JL, DM and JT conceived of the presented idea. PH, and MW summarized the reference and draft the manuscript. HG organized the figure and table. JL, DM and JT supervised the project and contributed equally to the final version of the manuscript. All authors contributed to the article and approved the submitted version.

## Funding

This work was supported by the National Natural Science Foundation of China (32170905, 81801550, 81630038 and 81971433);

## Conflict of Interest

The authors declare that the research was conducted in the absence of any commercial or financial relationships that could be construed as a potential conflict of interest.

## Publisher’s Note

All claims expressed in this article are solely those of the authors and do not necessarily represent those of their affiliated organizations, or those of the publisher, the editors and the reviewers. Any product that may be evaluated in this article, or claim that may be made by its manufacturer, is not guaranteed or endorsed by the publisher.
